# Sustained release delivery of favipiravir through statistically optimized, chemically cross-linked, pH-sensitive, swellable hydrogel

**DOI:** 10.1186/s40360-024-00752-8

**Published:** 2024-04-29

**Authors:** Arooj Khan, Muhammad Zaman, Muhammad Ahsan Waqar, Asif Mahmood, Talal Shaheer, Rai Muhammad Sarfraz, Kanwal Shahzadi, Azmat Ali Khan, Amer M. Alanazi, Milton Kumar Kundu, Md Rabiul Islam, Athanasios Alexiou, Marios Papadakis

**Affiliations:** 1 Faculty of Pharmaceutical Sciences, University of Central Punjab, Lahore, Pakistan; 2 Department of Pharmaceutics, Faculty of Pharmaceutical Sciences, Lahore University of Biological and Applied Sciences, Lahore, Pakistan; 3 Department of Pharmacy, University of Chakwal, Chakwal, Pakistan; 4 College of Pharmacy, University of Sargodha, Sargodha, Pakistan; 5 Faculty of Pharmacy, The University of Lahore, Lahore, Pakistan; 6 Pharmaceutical Biotechnology Laboratory, Department of Pharmaceutical Chemistry, College of Pharmacy, King Saud University, Riyadh, 11451 Saudi Arabia; 7 Pharmacy Discipline, Khulna University, Khulna, 9208 Bangladesh; 8 Department of Chemistry, Tennessee State University, 3500 John A Merritt Blvd, Nashville, TN 37209 USA; 9 University Centre for Research and Development, Chandigarh University, Chandigarh-Ludhiana Highway, Mohali, Punjab India; 10 Department of Research and Development, Funogen, Athens, Greece; 11 Department of Research and Development, AFNP Med, Wien, 1030 Austria; 12 Department of Science and Engineering, Novel Global Community Educational Foundation, Hebersham, NSW 2770 Australia; 13 Department of Surgery II, University Hospital Witten-Herdecke, University of Witten-Herdecke, Heusnerstrasse 40, 42283 Wuppertal, Germany

**Keywords:** Hydrogel, Sustained release, Polyethylene glycol, Favipiravir, Box-Behnken design

## Abstract

In the current work, favipiravir (an antiviral drug) loaded pH-responsive polymeric hydrogels were developed by the free redical polymerization technique. Box-Behnken design method via Design Expert version 11 was employed to furnish the composition of all hydrogel formulations. Here, polyethylene glycol (PEG) has been utilized as a polymer, acrylic acid (AA) as a monomer, and potassium persulfate (KPS) and methylene-bisacrylamide (MBA) as initiator and cross-linker, respectively. All networks were evaluated for in-vitro drug release (%), sol-gel fraction (%), swelling studies (%), porosity (%), percentage entrapment efficiency, and chemical compatibilities. According to findings, the swelling was pH sensitive and was shown to be greatest at a pH of 6.8 (2500%). The optimum gel fraction offered was 97.8%. A sufficient porosity allows the hydrogel to load a substantial amount of favipiravir despite its hydrophobic behavior. Hydrogels exhibited maximum entrapment efficiency of favipiravir upto 98%. The in-vitro release studies of drug-formulated hydrogel revealed that the drug release from hydrogel was between 85 to 110% within 24 h. Drug-release kinetic results showed that the Korsmeyer Peppas model was followed by most of the developed formulations based on the R^2^ value. In conclusion, the hydrogel-based technology proved to be an excellent option for creating the sustained-release dosage form of the antiviral drug favipiravir.

## Introduction

A sustained-release drug delivery system was intended to minimize toxicity, improve efficacy, and improve patient compliance by controlling the drug concentration at the target site [1]. Hydrogels are three dimensional pH responsive polymeric networks capable of imbibing larger volume of water in their interconnected voids. Upon exposure to a media of certain pH these systems undergo volume transitions due to chain relaxation as result of repulsion between prevalent functional groups in a system like –OH, –SO_3_H, –COOH and –NH_2_ etc [2].

Polyethylene glycol has various biomedical applications in drug delivery, wound dressing, and tissue engineering, because of its non-immunogenicity, biocompatibility, and resistance to the adsorption of proteins [3]. It is a synthetic polymer so its chemical and physical properties for example structure and chain length can easily be handled. It has non-cytotoxicity and powerful mechanical properties [4]. Due to the type, length, and ligand-binding properties of the hydrogel network, PEG-based hydrogels provide substantial benefits that are required for the modulation of drug carriers [5]. Acrylic acid (AA) is used as a monomer. For the preparation of hydrogel containing AA, the free radical polymerization technique is mainly used in which acrylic acid reacted with free radicals and electrophilic agents. Acrylic acid is cross-linked to form a hydrogel having a higher absorption capacity. It is combined with other polymers to produce diverse forms of pH-responsive hydrogels [6].

Methylene bis-acrylamide (MBA) is a crosslinking agent because of its two extremely carbon-carbon double bonds. It has capacbility to react with a variety of functional groups including –COOH, –NH_2_, and –OH while making a three-dimensional network [7]. Potassium sulfate (KPS) was used as an initiator (free radical generator) [8]. It is a white crystalline powder which can be simply dissolved in the water [9].

Favipiravir (FAV) is an antiviral drug which was permitted in 2014 in Japan against the influenza virus. It prevents viral replication by inhibiting RNA dependant RNA polymerase and is also used in COVID treatment [10]. The drug follows a very short half of 2 to 5.5 h. While the drug reaches to its maximum plasma concentration in 2 h followed by oral administration [11]. Current work aims to prepare a stable hydrogel system for oral delivery of Favipiravir using a free radical polymerization technique.

The objective of this study was to develop a dosage form, to enhance patient compliance as the drug appeared to have a very short half-life to avoid multiple dosing a sustained release formulation was developed. As hydrogels appeared to have better drug loading efficacy and drug release in a controlled manner to maintain the drug levels within the blood.

## Materials and methods

Favipiravir has been a gift from CCL Pharmaceuticals, Lahore, Pakistan. Polyethylene glycol 6000 (PEG), KPS, MBA as well as acrylic acid have been bought from Sigma Aldrich (United States). All other excipients used have been attained and distilled water was prepared in the research lab of UCP, Lahore-Pakistan.

### Formulations prepared by using a design expert

The hydrogel formulations of favipiravir were formulated by using the software of a design expert. In these trials following variables were included PEG as a polymer, AA as a monomer and MBA as a cross-linker. The amount of initiator was set constant (Table 1).

**Table 1:** Composition of PEG-6000 based hydrogel formulations by design expert

S. No	PEG 6000 (mmol) (X_1_)	Acrylic acid (mmol) (X_2_)	MBA (mmol) (X_3_)
1	0.0208	27.7546	0.1946
2	0.0208	34.6933	0.1297
3	0.0208	41.6319	0.1946
4	0.0208	34.6933	0.2594
5	0.0312	27.7546	0.1297
6	0.0312	27.7546	0.2594
7	0.0312	34.6933	0.1946
8	0.0312	34.6933	0.1946
9	0.0312	41.6319	0.2594
10	0.0312	41.6319	0.1297
11	0.0416	27.7546	0.1946
12	0.0416	34.6933	0.2594
13	0.0416	34.6933	0.1297
14	0.0416	41.6319	0.1946

### Development of PEG6000 co-poly(acrylate) hydrogels

By the free radical polymerization process, hydrogels were prepared. The PEG 6000 required amount was dissolved in distilled water while using a magnetic stirrer (Model, 78-1, Changzhou, China). Stirring was continued until the formation of clear solution. In a separate beaker required quantity of monomer and initiator (KPS) was added and mixed it well by constant stirring. Then this monomer solution was transferred into PEG solution dropwise and mixed it well with continuous stirring. After that cross-linker (MBA) was added in it with constant mixing on a magnetic stirrer. Finally, the distilled water to make the final volume (25 ml). Sonicator was used to remove traces of any dissolved oxygen from the polymerization solution. The final solution has been shifted into the test tube and placed into the water bath at 60 °C for 3 h until the hydrogel was solidified. After that, the test tubes were taken out of the water bath and left to cool to room temperature. Then cut the hydrogel into the shape of a cylindrical discs. The discs were washed by using a mixture of water and ethanol (70:30) to eliminate the unreacted material. Washing was continued until the stable pH reading of washing solution at pH meter.

**Fig. 1:**
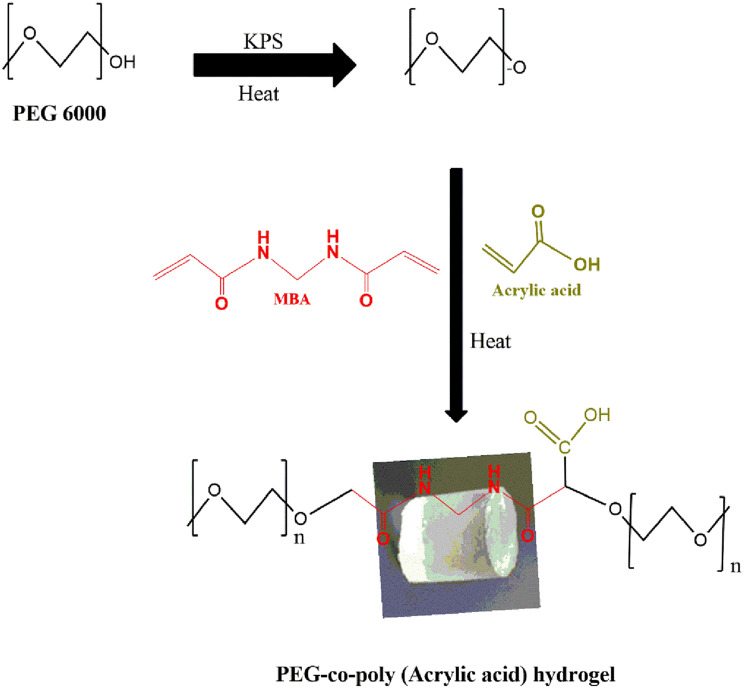
Proposed diagram of PEG-co-poly (acrylic acid) hydrogels

For drying purposes, washed discs were placed at 50 °C in an oven for 3 to 4 days. Then, the discs were removed from the oven as soon as they were dried [12]. The mechanism reaction of PEG/AA is specified in Fig. 1.

### Drug loading (%)

For drug loading, the method of post-loading was utilized. The 1% drug solution was prepared by dissolving 1 g of favipiravir in 100 ml ethanol while continuously stirring on a hot plate magnetic stirrer. pH of the drug solution was adjusted to 7.2 by adding triethanolamine drops. The dried disc of hydrogel was soaked in the drug solution until the constant weight was attained. The swollen hydrogel disc was removed from the solution and washed with distilled water to get rid of any residual drug mass from surface. The disc was then dried for 3 to 4 days in an oven at 40 °C until constant weight was achieved.

### Numerical optimization

In-vitro drug release (%), degree of swelling (%), sol-gel fraction (%), Porosity (%) and entrapment efficiency (%) were the responses studied by using a design expert. In current research work, the numerical optimization technique was adapted to optimize the process conditions. In this optimization approach, the desired goals were to improve drug loading efficiency, sustain the drug release to decrease the dosing frequency, optimum gel fraction (%), and decrease gel porosity (%) were the features to be controlled by using a design expert. Experimental values and predicted values were compared. Numerous trials of formulations were prepared, and depending upon the desired outcomes optimized formulation was selected. The swelling at pH 1.2 was found within the desirable range i.e. negligible. The desirability of the optimized formulation was 0.953 and the composition consisting of PEG was 0.625 g, MBA was 14.23 g and acrylic acid was 0.138 g.

### Characterization of hydrogels

The developed hydrogels were evaluated for different parameters i.e. organoleptic properties, swelling studies, porosity measurements (%), sol-gel fraction (%), entrapment efficiency, in-vitro drug release, SEM, FTIR and PXRD studies.

#### Organoleptic evaluation

Hydrogel formulation homogeneity, shape, and color were all visually observed.

##### Shape, color, and homogeneity

The shape and color of hydrogels were visually observed. For the detection of surface homogeneity and any other kind of abnormality like aggregation, discoloration, or spotting, hydrogels were inspected visually.

#### Swelling studies

The hydrogels swelling ratio were studied by using different pH buffer solution which includes pH 1.2, pH 6.8, and pH 7.2 in order to predict network’s behavior when exposed to different pH levels within the GIT. Moreover, to ensure sustained drug release potentials at higher pH values and restricted or very low drug release at lower pH value swelling studies were executed. These studies were continued until a constant weight was achieved, dried hydrogel discs were dipped in different buffer solutions for up to about 72 h. At a predetermined time intervals,swollen hydrogels were taken out from the buffer solution and their surface was made dried by using filterpaper and reweighed. The swelling ratio of the hydrogel can be calculated by using following equation:

1 $${\text{Swelling}\,\text{ratio}} = \frac{{\left[ {{\text{Ws}} - {\text{Wd}}} \right]}}{{{\text{Wd}}}} \times 100$$

In the Eq. (1), “Ws” is the weight of swollen hydrogel and “Wd” is the dry hydrogel weight [13].

#### Sol-gel fraction

In distilled water, hydrogel discs (dried) was soaked for up to 48 h at 37 °C. In an oven, the swollen hydrogel disc was dried at 50 °C till a constant weight was attained The gel fraction and solfraction were calculated by using following equation:

2 $${\text{Sol}\,\text{fraction\,}}\left( {\text{\% }} \right) = \frac{{\left[ {{\text{Wo}} - {\text{Wi}}} \right]}}{{{\text{Wo}}}} \times 100$$

3 $${\text{Gel}\,\text{fraction\,}}\left( {\text{\% }} \right) = 100 - {\text{Sol}\,\text{fraction}}$$

Where, W_o_ is the hydrogel weight before extraction and W_i_ is the hydrogel weight after extraction [14].

#### Measurement of porosity (%)

For porosity measurement, a solvent replacement method was used. In this method, dried hydrogel was soaked in ethanol overnight for 24 h. After 24 h, hydrogel discs were blotted for the removal of extra ethanol and then were weighed. Porosity of hydrogel was calculated by the using following equation [15];

4 $${\text{Porosity}} = \left( {{\text{M}}2 - {\text{M}}1} \right)\,{{\rho V\,}} \times 100$$

Where, *M*1 is hydrogel mass before dipped in ethanol, *M*2 is hydrogel mass after dipped in ethanol, *ρ* is the absolute ethanol density and *V* is the hydrogel volume.

#### In vitro drug release studies

This test was performed on the USP dissolution Type II paddle apparatus. The drug loaded hydrogel discs were placed in baskets containing dissolution medium (900 mL) The dissolution medium consist of a phosphate buffer of pH 1.2 and 6.8. The apparatus was operated at 50 rpm speed and temperature was set at 37 °C. At a distinct intervals of time, the 5 mL sample was removed and replaced with the fresh media. The absorbance was checked by using UV/Vis spectrophotometer at 233 nm.

The equation derived via the calibration curve was utilized for calculating the amount of drug released at each time point [16]. The drug release (%) was calculated by the following equation;

5 $$\eqalign{\% \,\,Drug\,Release\, &= \,Amount\,of\,drug\,released\,\cr &\quad/Amount\,of\,drug\,added\, \times \,100}$$

#### Entrapment efficiency (%)

Drug-loaded hydrogel disc was immersed in 50 mL ethanol for 24 h and was allowed to swell. After that, the swollen hydrogel disc was crushed in a pestle and mortar, and crushed hydrogel disc particles were transferred to the same ethanol solution. The mixture was then homogenized at 13,000 rpm for 2 min at a magnetic hot plate (ATO-HS-12, Golden Springs DR. I, Diamond Bar, USA). The homogenized mixture was then centrifuged for 5 min and filtered it. Through a UV Spectrophotometer absorbance of the solution was checked at 233 nm wavelength. Using the favipiravir drug calibration curve and absorbance measurement, the total amount of drug recovered was then calculated. The entrapment efficiency (%) was calculated by using following equation [17];

$$\eqalign{&Entrapment\,Efficiency\,\left( \% \right)\cr &= \frac{{Total\,amount\,of\,drug\,recovered}}{{Total\,amount\,of\,drug\,added}} \times \,100}$$

#### Drug release kinetics

For determining, formulation drug release pattern, release kinetics have been assessed by the use of software DD solver, and R^2^ values for Zero order, First Order, Korsmeyer–Peppas model, Higuchi model, and Hixson–Crowell model were calculated to explore best fit kinetic model [18].

**Zero order equation:**

6 $$Mt = Mo - Kot$$

Here: k_o_ represents release rate constant and t is the time

**First-order equation:**

7 $$Mt = 1 - {e^{ - {K_1}}}t$$

From Eq. (7), k_1_ stands for the release rate constant.

**Higuchi’s equation**

8 $$Mt = {K_h}{t^{1/2}}$$

k_H_ signifies for Higuchi release rate constant.

**Hixson-Crowell equation**

9 $${\text{F = 100 }} \times {\text{ [1 - (1 - }}{{\text{k}}_{{\text{HC}}}}{\text{ }} \times {\text{ t}}{{\text{)}}^{\text{3}}}{\text{]}}$$

From Eq. (9), k_HC_ represents the Hixson-Crowell constant.

Furthermore, for a better description of the mechanisms of drug release, Korsmeyer-Peppas model has been applied:

10 $$\frac{{{M_t}}}{{{M_\infty }}} = K{t^n}$$

Where, k_KP_ is a constant value representing the geometrical and structural properties of device while, “n” represents release exponent that represents the mechanism of release of drug [18]. DD solver software was used for the evaluation of kinetic models and the values of R^2^ were measured for each model.

#### FTIR analysis

The FTIR spectra of KPS, MBA, AA, PEG, and Favipiravir, a drug-unloaded disc, as well as a drug-loaded disc of hydrogel were recorded. By using the ATR-FTIR the samples of hydrogels were scanned and compatibility of ingredients with each other and development of new network was also confirmed.

#### X-rays diffraction

X-ray diffractometer was used to conduct the X-ray diffraction on the drug-loaded PEG hydrogel and favipiravir pure drug, with scanning range from 10° to 45°. To determine the constituent’s nature i.e. crystalline or amorphous, X-ray diffraction study was used.

#### Scanning electron microscopy (SEM)

Scanning Electron Microscopy (SEM) with EDX and E-beam Lithograph FEI Nova 450 Nano SEM machine was used. SEM has been done for studying the morphology as well as size of the formulation of hydrogel. Surface of hydrogel has been examined via SEM [19].

## Results and discussions

### Swelling studies

PEG/AA hydrogel swelling in all trials was done at pH 7.2, 1.2, and 6.8. In all formulations, the initiator (KPS) amount was the same but the monomer, cross-linker, and oligomer amounts were different. On swelling the impact of changing concentration of the cross-linker, monomer, and PEG was studied.

**Fig. 2:**
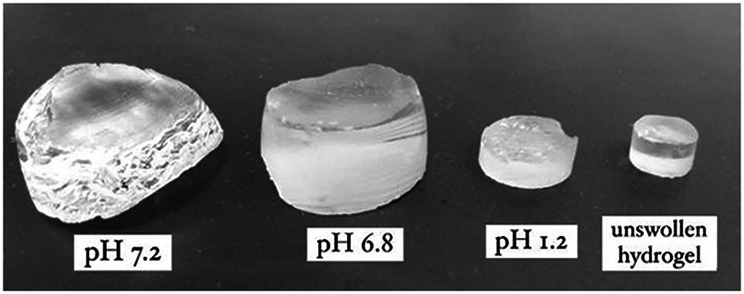
Swelling studies of PEG hydrogels in pH 6.8, 7.2 and 1.2

The hydrogel exhibited more swelling at pH 6.8 as compared to pH 1.2 (Fig. 2). Hydrogel having AA showed a low degree of swelling at gastric pH, however when they passed into the GIT, the swelling increases as increases in pH. Acrylic acid contains a carboxyl group its structure and swelling of hydrogel was affected because of the presence of the ionized carboxylate ions at higher pH. PEG contains (–OH) groups, which make it highly appealing to water and impart it hydrophilic character. It was seen that at higher pH carboxylic groups get ionized due to which electrostatic repulsive forces prevail between polymeric chains and results in expansion of the developed network [20].

#### Cross-linker (MBA) impact on hydrogel swelling

Crosslinking agent concentration is a major parameter in hydrogel because it directly affects the swelling property. As we increase the crosslinker concentration, a decrease in the hydrogel swelling was recorded. Because of a higher amount of crosslinking agent, the hydrogel’s structure turned compact, dense and reduced pore sizeresulted in poor water absorption.

#### Monomer effect on hydrogel swelling

Results exhibited that the swelling of hydrogel was increased as we increase the amount of monomer due to availability of excessive carboxylic acid groups. As presented by Sindhu et al., because of carboxylic group ionization at higher pH by increased monomer concentration, consider raised in swelling was observed [21]. Due to availability of higher ionized carboxylic groups upon incremental rise in AA contents, swelling was pronounced leading to higher uptake of swelling media. In acrylic acid addition, the amount of inorganic compound/hydrophilic polymer increased.

### Sol-gel fraction (%)

Gel fraction determination was done for checking the quantity of uncrosslinked ingredients which endured into the hydrogel. Gel fraction (%) of PEG-based hydrogels ranged from 88.1 to 99.3%. As we have seen that by raising the amount of cross-linker, polymer and monomer there was an enhancement in hydrogel gel fraction (%). The same result showed in the work of Barkat et al., raised in the percentage of gel fraction was seen because of the high concentration of polymer, cross-linker, and monomer. In higher concentrations of monomer, there was the presence of primary radicals [22].

### Porosity (%)

Porosity test was executed to analyze porous structure of hydrogel. The porosity of PEG hydrogel was ranging from 10.5% to 85.19 %. The rise in porosity (%) was seen by the increasing the quantity of monomer. On the other hand, by increasingthe amount of crosslinker (MBA) porosity (%) was decreased (that might be because of an enhanced crosslink density) [23]. In LSH-co-MAA formulations comparable results have been observed by Shabir et al. As increased in the amount of MBA a decrease in porosity occurred [24].

### Entrapment efficiency of drug (%)

Drug entrapment in PEG hydrogels was ranging from 82.13 to 99.8%. As we have seen that by increasing in the amount of monomer and polymer, efficiency of drug entrapment was increased. A comparable result was seen in work by Nautiyal et al., as an increased quantity of polymer as well as monomer the effectiveness of drug entrapment was increased [17]. By increasing MBA contents drug entrapment efficiency was reduced because of higher crosslinking density leading to formation of denser network thus promoting poor uptake of drug solution into the network. In their article, Malik et al., reported on similar work in which they created a hydrogel using CS/XG [25].

### In-vitro drug release studies

PEG drug-loaded hydrogel’s drug release studies have been carried out through the Dissolution apparatus (USP). Drug release studies of PEG hydrogels range from 89.13 to 118.0%. Similar work was reported by Ramadan et al., in which hydrogel drug release depends on the concentration of monomer, polymer, and cross-linker [26].

#### PEG concentration effect on the in-vitro drug release

Results presented that as increased in PEG (Formulations F3, 5, 9, and 10) concentration the rise in drug release was noticed. It is evident from the results that as the concentration of polymer in formulation increases a large hydrogel network was observed. The possible reason was that the large network diffuses more drugs from the hydrogel network and improves drug release [27]. Same results have been seen by Dong et al., worked who created dual cross-linked hydrogel in which polymer concentration increased drug release [28].

#### Cross-linker and monomer concentration effect on in-vitro drug release

As seen by increasing the quantity of the monomer enhanced hydrogel drug released was increased from hydrogel. In hydrogel, the greater amount of crosslinker decreased drug release. Same results reported by Bueno et al. worked who created hydrogel based on GG/PVP co-polyacrylic acid. It was shown that the drug release increased as the concentration of acrylic acid increased. Increased cross-linker concentrations reduce linked pores between the monomer and polymer, which reduces fluid medium penetration and, thus, reduces diffusion [29].

### Drug release kinetics

By using diverse kinetic models (zero order, first order, kosmeyer Peppas model, Higuchi model Hixson-Crowell) with the DD Solver software and their R^2^ values the design of drug release at pH 6.8 of P.B was observed.

All formulations containing PEG followed the Korsmeyer–Peppas model. The R^2^ value of the Korsmeyer–Peppas model implicated that the drug concentration being released from the gel was uniform regarding time. Similar research was done by Rahmani et al., where hydrogels of diclofenac sodium were formulated, in which all of the formulations showed th Koesmeyer Peppas model as the best-fit model for drug release kinetics [30]. The optimized formulation (F15) of PEG hydrogel has also shown that best fit model was Korsmeyer–Peppas. Mechanism of the drug release has been Fickian diffusion if its exponent ‘n’ value ≤ 0.5. “n” value of all PEG formulations was ≤0.5 that ratified Fickian type of diffusion mechanism of drug release [31].

### Mathematical modeling

This method was done to find out the uncertainties present in observed data. This test has been executed to illustrate the selected model as well as evaluate its capability for evaluation of formulated hydrogels. A quadratic model was selected as well as mathematical modeling has been done through the software of a design expert. A software design expert was chosen to calculate the responses.

Polynomial Equation:

11 $$\eqalign{ {\text{Y }} &= {{\text{X}}_{\text{1}}} - {{\text{X}}_{\text{2}}} + {{\text{X}}_{\text{3}}} + {{\text{X}}_{\text{1}}}{{\text{X}}_{\text{2}}} \cr &\quad + {\text{ }}{{\text{X}}_{\text{1}}}{{\text{X}}_{\text{3}}} - {{\text{X}}_{\text{2}}}{{\text{X}}_{\text{3}}} + {{\text{X}}_{\text{1}}}^{\text{2}} - {{\text{X}}_{\text{2}}}^{\text{2}} + {{\text{X}}_{\text{3}}}^{\text{2}}}$$

#### Response 1: degree of swelling (%)

12 $$\eqalign{ {\text{Swelling}\,\text{at}\,\text{pH}}\,{\text{6.8 }} &= + 159.02 + 83.47 - 6.78 \cr&\quad + 159.05 - 128.81 - 729.70 \cr&\quad + 266.85 - 580.02 + 700.08}$$

**Fig. 3:**
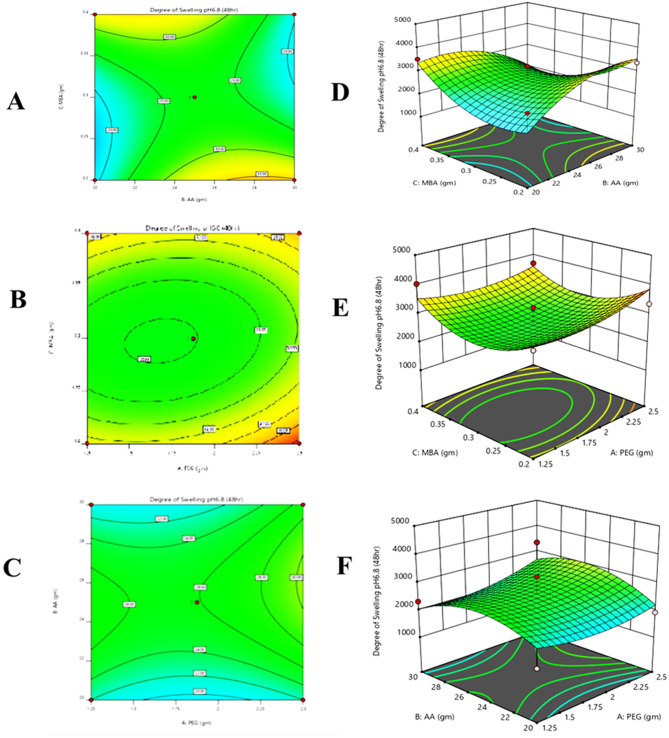
All pilot formulations of PEG hydrogels. (**A**) contour and (**D**) 3D graph between monomer and cross-linker, (**B**) contour and (**E**) 3D graph between polymer and monomer, (**C**) contour and (**F**) 3d graph between polymer and cross-linker were drawn to determine their effect on swelling

It was observed from the contour, 3D graph, and polynomial equation that complete response has been constructive. X_1_ and X_2_ variables’ positive values indicated that swelling increased and X_3_ variable negative value indicated that swelling decreased (Fig. 3).

#### Response 2: gel fraction (%)

13 $$\eqalign{ {\text{Gel}\,\text{Fraction}}\,\left( {\text{\% }} \right) &= + 0.7250 + 0.2500 + 0.9250 \cr&\quad - 0.5500 + 0.8500 - 2.40 \cr&\quad+ 0.6250 - 1.37 - 3.68}$$

**Fig. 4:**
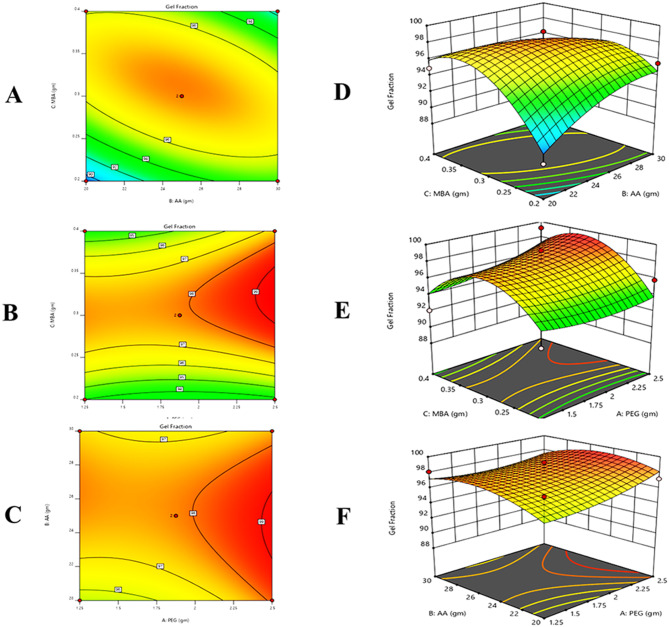
All pilot formulations of PEG hydrogels. (**A**) contour and (**D**) 3D graph between monomer and cross-linker, (**B**) contour and (**E**) 3D graph between polymer and monomer, (**C**) contour and (**F**) 3d graph between polymer and cross-linker were drawn to determine their effect on gel fraction

It was observed from the contour, 3D graph, and polynomial equation that complete response has been constructive. X_1,_ X_2,_ and X_3_ variables’ positive values indicated that gel fraction increased (Fig. 4).

#### Response 3: porosity (%)

14 $$\eqalign{ {\text{Porosity}}\,\left( {\text{\% }} \right) &= + 20.10 + 17.38 - 6.44 \cr&\quad + 4.51 - 20.12 + 4.11 \cr&\quad+ 8.68 + 34.20 + 53.71}$$

**Fig. 5:**
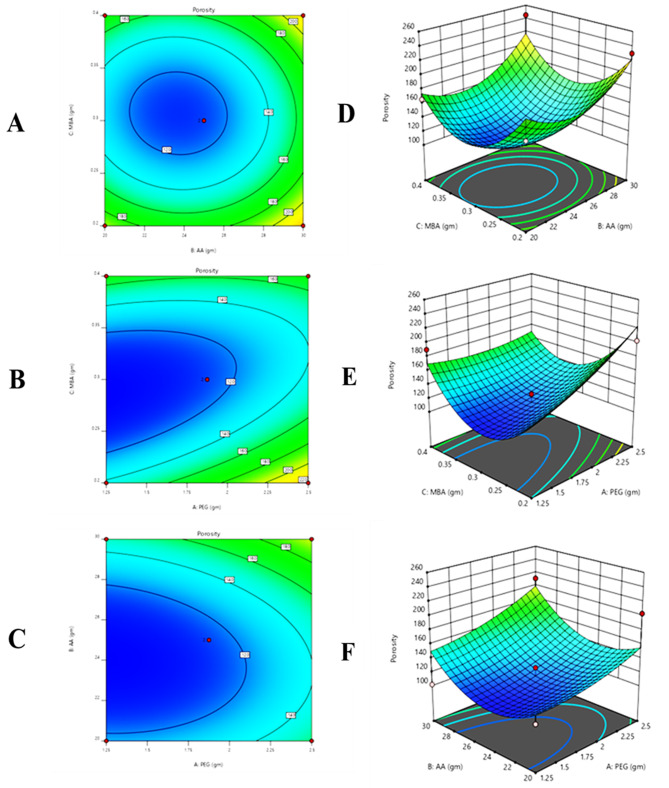
All pilot formulations of PEG hydrogels. (**A**) contour and (**D**) 3D graph between monomer and cross-linker, (**B**) contour and (**E**) 3D graph between polymer and monomer, (**C**) contour and (**F**) 3d graph between polymer and cross-linker and polymer were drawn to determine their effect on the porosity

It was observed from the contour, 3D graph, and polynomial equation (Eq. 14) that complete response has been constructive. X_1_ and X_3_ variables positive values indicated that porosity increased and X_2_ negative values indicated that porosity decreases (Fig. 5).

#### Response 4: entrapment efficiency (%)

15 $$\eqalign{ {\text{EE\% }} &= + 2.45 + 0.3512 - 2.02 - 4.86 \cr&\quad - 0.1450 - 4.06 + 0.3087 - 4.28 + 0.4087}$$

**Fig. 6:**
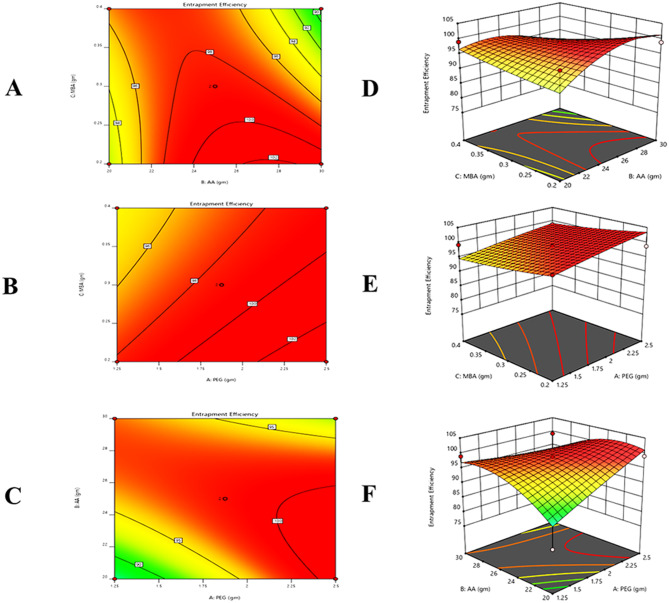
All pilot formulations of PEG hydrogels. (**A**) contour and (**D**) 3D graph between monomer and cross-linker, (**B**) contour and (**E**) 3D graph between polymer and monomer, (**C**) contour and (**F**) 3d graph between polymer and crosslinker were drawn to determine their effect on the drug entrapment

It was observed from the contour, 3D graph, and polynomial equation (Eq. 15) that complete response has been constructive. X_1_ and X_2_ variables positive values indicated that drug entrapment increased and X_2’s_ negative values indicated that drug entrapment decreases (Fig. 6).

#### Response 5: in vitro drug release (%)

16 $$\eqalign{ {\text{Drug}\,\text{release}} &= + 4.97 + 0.2012 - 1.56 - 1.85 \cr&\quad - 2.07 + 0.2075 - 2.27 - 3.73 - 6.64}$$

**Fig. 7:**
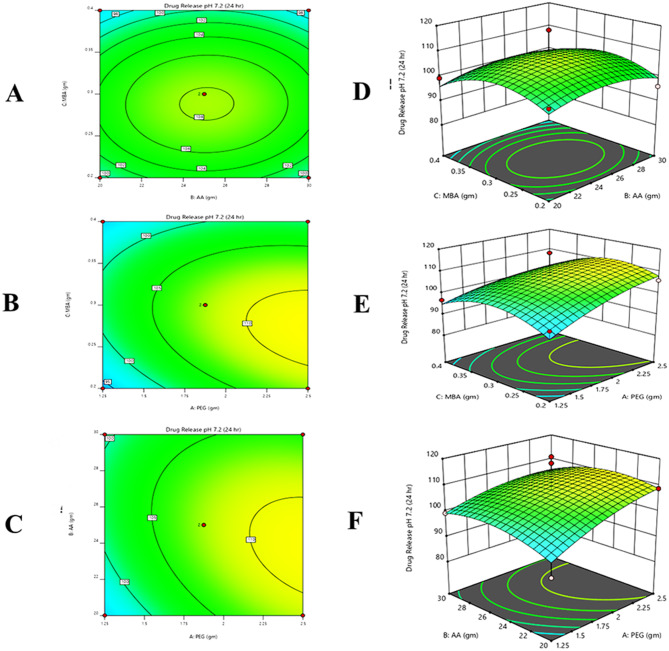
All pilot formulations of PEG hydrogels. (**A**) contour and (**D**) 3D graph between monomer and cross-linker, (**B**) contour and (**E**) 3D graph between polymer and monomer, (**C**) contour and (**F**) 3d graph between polymer and crosslinker were drawn to determine their effect on the drug release

It was observed from the contour, 3D graph, and polynomial equation (Eq. 16) that complete response has been constructive. X_1_ and X_2_ variables positive values indicated that drug release increased and X_2’s_ negative values indicated that drug release decreases. The positive value of X_1_, and X_2_ suggested that this variable would increase study response to drug release but negative value of X_2_ has demonstrated that by enhancing value of X_2_ we could minimize the drug release (Fig. 7).

### Numerical optimization

Predicted results of evaluation parameter of optimized formulation (PEG hydrogel) have been compared with results obtained. Predicted results were obtained by using Design Expert software. Outcomes of hydrogel that has been optimized, had shown no major difference from the predicted results. Optimized formulation of PEG hydrogel succeeded because they contain all the necessary characteristics which include swelling studies, gel fraction, in-vitro drug release, porosity as well as entrapment efficiency. Table 2 shows a comparison of obtained results and predicted outcomes.

**Table 2:** Comparison of the obtained results and predicted outcomes of PEG hydrogels

Parameter	Predicted outcomes	Obtained results
Porosity	127.336%	85.19%
Gel fraction	97.812%	96.20%
Entrapment efficiency	98.654%	98.85%
Degree of swelling pH 6.8	2104.747%	1914%
Degree of swelling pH 7.2	2500.001%	2341%
Degree of swelling pH 1.2	128.607%	140%
In-vitro drug release	100.000%	99.25%

### Fourier-transform infrared spectroscopy (FTIR)

**Fig. 8:**
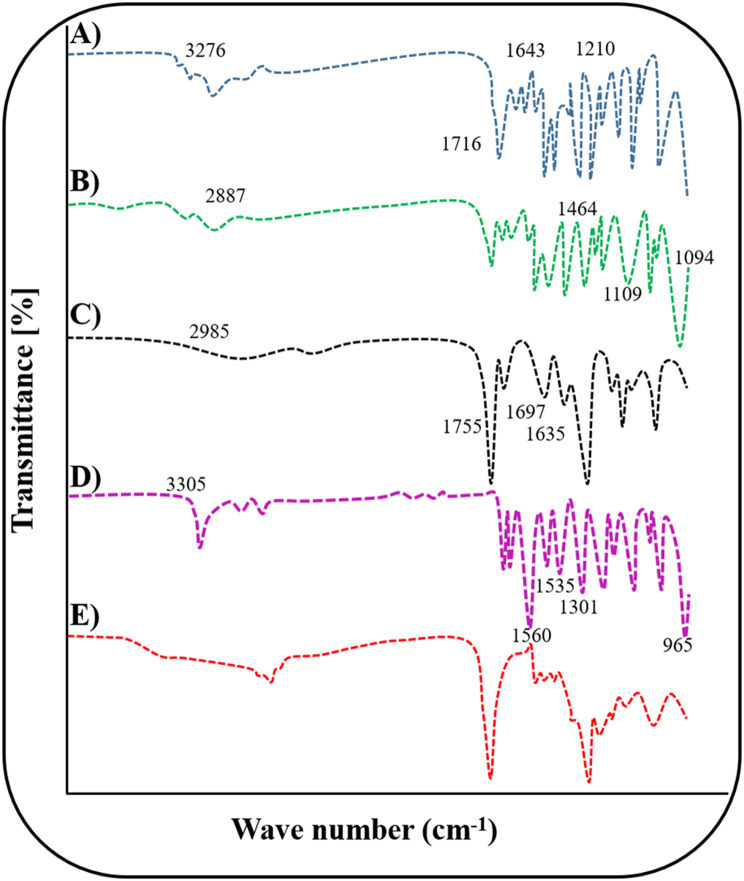
Representing the FTIR analysis of (**A**) favipiravir, (**B**) PEG 6000, (**C**) AA, (**D**) MBA (**E**) PEG-co-poly (acrylic acid) hydrogel

To evaluate the occurrence of functional groups in hydrogel, we did the FTIR (Fig. 8). The pure drug FTIR spectrum showed numerous peaks.

The FTIR spectrum was used to indicate the active constituent’s functional groups constructed on IR spectrum peak values. FTIR spectra of favipiravir, PEG 6000, AA, MBA, KPS, drug-loaded and unloaded hydrogels disc of both polymers were conducted.

Pure favipiravir drug had shown various peaks in FTIR spectrum (Fig. 8A). At 3276 cm^−1^ one peak was observed of –OH stretching. At 1716 cm^−1^ the toughest peak was observed linked to the C=O stretching. At 1643 cm^−1^ peaks were observed C=N stretching. At 1210 cm^−1^ peaks were observed are corresponded to C–F stretching [32]. In this spectrum of PEG 6000 highest peaks were observed of C–H stretching at 2887 cm^−1^ (Fig. 8B). C–H bending peak has been detected at 1464 cm^−1^. Band detected at 1094 cm^−1^ and 1109 cm^−1^ are attributed towards stretching of C–O–H and C–O–C [33].

In the Acrylic acid spectrum (Fig. 8C) at 2985 cm^−1^ the peak was observed of the –OH bond. At 1755 cm^−1^ the band was observed linked to the -the COOH group. The C=C and C=O stretching peaks were observed at 1635 cm^−1^ and 1697 cm^−1^ [34]. In the KPS spectrum at 1385 cm^−1^ one major peak was observed corresponding to S=O stretching [35]. In the MBA spectrum (Fig. 8D) at 3305 cm^−1^ a noticeable peak was observed that revealed the N–H stretching while at 1560 cm^−1^ was seen that linked to C=O stretching vibrations as well as at 1535 cm^−1^ demonstrated N–H distortion. C–N stretching vibrations are displayed by the peak at 1301 cm^−1^. At 965 cm^−1^ peaks were observed linked to N–C bond stretching vibrations and at 955 cm^−1^ peak exhibited C–Cα stretching.

These same peaks could be seen in the hydrogel disc that was drug loaded, indicating that the drug was well-matched with other elements. Disappearance of peaks, strengthening peak intensities, shifting of peaks and emergence of new peaks confirmed formation of a new network (Fig. 8E). There was no drug-component conflict with the hydrogel’s other elements.

### X rays diffraction (XRD)

**Fig. 9:**
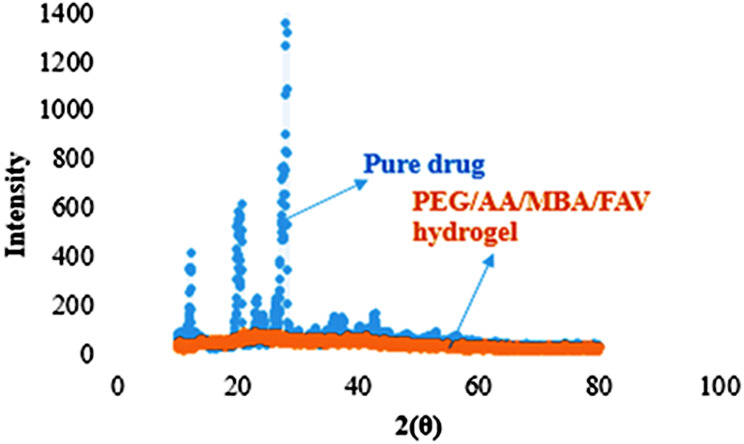
XRD diffractograms of favipiravir and formulation code

The favipiravir and drug-loaded hydrogel diffraction patterns were compared. The favipiravir drug’s intense and sharp peaks were noted at an angle of 28°, indicating that the drug nature was crystalline. These scattered peaks demonstrated the drug’s amorphous presence in the hydrogel formulation. The underlying crystal structure of the PEG was altered, and high crystalline peaks with high intensity were swapped out for low-intensity peaks, demonstrating successful polymer grafting (Fig. 9).

Pure drug XRD had shown intense as well as sharp peaks, representing a crystalline structure, nonetheless, drug-loaded hydrogel XRD shows no crystalline structure and sharp peaks.

### Scanning electron microscopy (SEM)

**Fig. 10:**
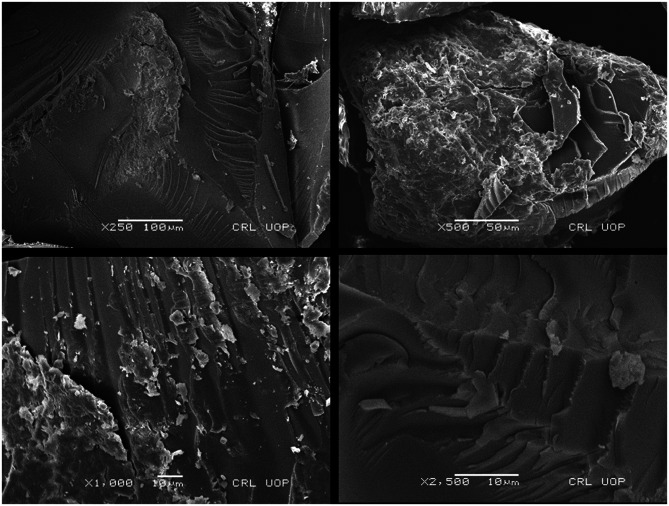
SEM photomicrograph of PEG6000 co-poly(acrylate) hydrogels

The SEM analysis was performed on Favipiravir loaded PEC-co-poly (acrylic acid) hydrogels to explore surface morphology of the developed network. SEM photomicrographs revealed compact denser morphology with wavy surfaces. Visible cracks along with pores were also recorded at maginification 500× and 1000× (Fig. 10). Existence of cracked surfaces facilitate update of swelling and dissolution media and hence promote pronounced swelling of the network and release incorporated Favipiravir at favourable pH. Ahmed et al. detected the same outcomes. They prepared a hydrogel based on β-CD to increase solubility of acyclovir.

These findings suggested that there was an existence of weak bonding between AA and PEG that leads to higher swelling in their polymeric network. In Fig. 10, small cracks were seen, those might be due to the gel dehydrating and partially collapsing. Deprotonation of carboxylic acid functional group is the responsible factor for expansion of polymeric network as a result of emergence of repulsive forces between deprotonated carboxylate ions (Table 3).

**Table 3:** Comparison between current work and relevant previous studies

Sr. No.	Previous studies	References
1	Thermally stable venlafaxine HCl loaded polyethylene glycol-co-poly (acrylic acid) hydrogels were developed using benzyl peroxide as initiator. Equilibrium swelling was investigated at pH 1.2 and pH 7.5. Venlafaxine HCl release was 33% and 91% at pH 1.2 and pH 7.5	[36]
2	Gamma radiation assisted development of polyethylene glycol-co-poly (acrylic acid) hydrogels for site specific delivery at a controlled rate. Optimum swelling i.e. more than 2500% documented at pH 7 instead of pH 1. Significant drug release was recorded in at elevated pH levels	[37]
3	Ultraviolet radiation assisted development of biocompatible polyethylene glycol –co- poly (acrylic acid) interpenetrating network	[38]
4	Aqueous free radical polymerization technique was employed to develop thermally stable PEG based hydrogels for controlled delivery of Ivabradine hydrochloride. Drug release and swelling kinetics were conducted at pH 1.2 and pH 6.8	[39]
5	Thermally stable, biocompatible, mechanically strong and non-cytotoxic PEG-co-poly (acrylic acid) hydrogels developed for diffusion based absorption purposes. Swelling studies were conducted at pH 2, 7 and 10. Pronounced swelling reported at pH 7 and pH 10	[40]
6	PEG-co-poly (acrylic acid) hydrogels developed via free radical solution UV polymerization. Theophylline release was higher at lower crosslinker content. Release was pH dependent	[41]

## Conclusions

Research goal was successfully attained because favipiravir hydrogels with better drug loading capacity and the capability to deliver the drug continuously for 24 h have been created and evaluated. The use of statistical technique appeared to be beneficial because it not merely aided in evaluation and design of hydrogel formulation nonetheless also in its optimization. Experimental work has been determined to be repeatable because results of optimized formulation have been similar to those that the design expert had predicted for optimal formulation. Thus, it could be established that drug delivery system of hydrogel has been efficient at the loading the higher drug dose as well as for the loaded APIs sustained release In addition, the design expert would be a helpful tool for time as well as money savings by decreasing the need for pointless trials for formulation optimization.

## Data Availability

All data generated or analysed during this study are included in this published article.
